# Platelet role in the prediction of MIS-C severity

**DOI:** 10.3389/fped.2023.1153623

**Published:** 2023-06-08

**Authors:** Ausra Snipaitiene, Aurelija Sirataviciene, Leila Varoneckaite, Rima Sileikiene, Lina Jankauskaite

**Affiliations:** ^1^ Pediatric Department, Medical Academy, Lithuanian University of Health Sciences, Kaunas, Lithuania; ^2^ Pediatric Department, Hospital of Lithuanian University of Health Sciences Kaunas Clinics, Kaunas, Lithuania; ^3^ Institute of Physiology and Pharmacology, Lithuanian University of Health Sciences, Kaunas, Lithuania

**Keywords:** multisystem inflammatory syndrome in children, COVID-19, biomarker, children, platelets

## Abstract

**Introduction:**

Multisystem inflammatory syndrome in children (MIS-C) has been reported as one of the cytokine storm syndromes associated with COVID-19. Despite the several proposed diagnostic criteria, MIS-C remains a diagnostic and clinical challenge. Recent studies have demonstrated that platelets (PLTs) play a crucial role in COVID-19 infection and its prognosis. This study aimed to investigate the clinical importance of PLT count and PLT indices in predicting MIS-C severity in children.

**Patients and methods:**

We conducted a retrospective single-center study at our university hospital. A total of 43 patients diagnosed with MIS-C during a 2-year period (from October 2020 to October 2022) were included in the study. MIS-C severity was evaluated according to the composite severity score.

**Results:**

Half of the patients were treated in the pediatric intensive care unit. No single clinical sign was associated with a severe condition, except for shock (*p* = 0.041). All the routine biomarkers, such as complete blood count (CBC) and C-reactive protein (CRP), used for MIS-C diagnosis were significant in predicting MIS-C severity. Single PLT parameters, such as mean PLT volume, plateletcrit, or PLT distribution width, did not differ between the severity groups. However, we found that a combination of PLT count and the previously mentioned PLT indices had the potential to predict MIS-C severity.

**Conclusions:**

Our study emphasizes the importance of PLT in MIS-C pathogenesis and severity. It revealed that together with routine biomarkers (e.g., CBC and CRP), it could highly improve the prediction of MIS-C severity.

## 1. Introduction

During the COVID-19 pandemic, severe acute respiratory syndrome coronavirus (SARS-CoV-2) infection in children was and is related to a less severe disease compared with that in adults. However, some children also develop COVID-19-related sequelae. Multisystem inflammatory syndrome in children (MIS-C) has been reported as one of the cytokine storm syndromes associated with COVID-19 and also in most of the patients who partially or fully meet the criteria of Kawasaki disease (KD) (1). Despite the several proposed diagnostic criteria (2–5), MIS-C remains a diagnostic and clinical challenge. This is evident in the variety of clinical MIS-C presentations, which can mimic different conditions from macrophage activation syndrome (MAS)-like features to less severe disease phenotypes, contributing to diagnostic complexity (6). The main symptoms of MIS-C, such as fever, rash, and signs of systemic inflammation resulting in multisystem organ dysfunction, can be found in many other infectious or inflammatory diseases in children (7). Nevertheless, a clear relationship between MIS-C outbreaks and previous SARS-CoV-2 infections defines it as a postinfectious condition (8–12). In the last few years, MIS-C has been scored according to its severity. Some patients demonstrate mild systemic symptoms and prolonged fever, while others present with signs of cardiac injury and shock, leading to admission to a pediatric intensive care unit (PICU) (6, 11). To date, efforts are ongoing to understand the pathogenesis of this condition and analyze any biomarkers that could predict the severity and outcomes of the disease.

One of the pathogenesis arms in severe SARS-CoV-2 infection and MIS-C is the activation of coagulation, resulting in a prothrombotic state in severe patients (13–15). The role of activated PLTs in the stimulation of immune cells and their interaction with different pathogens has been well described in several studies (16–18). For example, PLTs are involved in the formation of neutrophil (Neu) extracellular traps and in phagocytosis (19, 20). PLT counts and PLT indices, such as mean PLT volume (MPV) and PLT distribution width (PDW), which are easily obtainable from routine blood samples, are widely discussed as biomarkers and prognostic factors in severe bacterial infections, such as sepsis (21, 22). Moreover, they are promising markers for distinguishing viral infections, such as flu and COVID-19 (23). In addition, recent studies have demonstrated that PLTs play a crucial role in COVID-19 infection and its prognosis (24, 25). Only a few studies discuss PLT count as a risk factor for MIS-C and its severity (26–29). Thus, we aimed to investigate the clinical importance of PLT count and PLT indices in predicting MIS-C severity in children who presented to our university hospital and were diagnosed with MIS-C.

## 2. Materials and methods

### 2.1. Study design and study population

This retrospective single-center study was conducted at the Hospital of Lithuanian University of Health Sciences Kauno Klinikos. Data of all patients who met the World Health Organization (WHO) case definition of MIS-C (4) referred and treated from 1 October 2020 to 1 October 2022 were analyzed.

### 2.2. Data collection

Demographic data (age, gender), living location, past medical history, and data regarding previous COVID-19 infection (infection, contact, and duration from the contact) were collected from an electronic data system. The day of contact with the virus was defined according to the positive epidemiological anamnesis of confirmed COVID-19 illness in close relatives of the same household or school or other close contacts. Moreover, we included admission to the PICU, total length of stay (LOS) in the ward, and LOS in the PICU in the data analysis. In addition, MIS-C severity was evaluated according to the composite severity score proposed by Brisca et al.—Gaslini severity assessment tool (GSATool) (30). The groups were defined according to clinical presentation, signs of cardiac dysfunction on echocardiography, increased cardiac enzyme levels, and signs of laboratory features of MAS (30). Instead of using four severity classes as described in the original study, because of the small sample size of the study, we divided the patients into two groups: Group 1 (less severe patients corresponding to classes I and II according to the GSATool) and Group 2 (severe patients representing classes III and IV of GSATool) (30).

### 2.3. Laboratory tests

Considering the presentation signs of MIS-C, routine markers as follows were obtained: complete blood count (CBC), C-reactive protein (CRP), erythrocyte sedimentation rate (ESR), procalcitonin (PCT**), lactate dehydrogenase (LDH), albumin, creatine, urea, alanine transaminase (ALT), aspartate aminotransferase (AST), gamma-glutamyl transferase (GGT), cardiac dysfunction markers [troponin I, NT-proB-type natriuretic peptide (pro-BNP)], evidence of coagulopathy [prothrombin time (PT), activated partial thromboplastin time (APTT), international normalized ratio (INR), fibrinogen (FB), and elevated D-dimer (DD) levels], and signs of possible macrophage activation [ferritin (FR)]. The results of all the mentioned biomarkers were included in the data analysis. From the initial CBC, white blood cell (WBC), Neu, lymphocyte (Lymph), PLT count, hemoglobin (Hgb), MPV, plateletcrit (PCT), and PDW data were collected.

### 2.4. Statistical analysis

Statistical analysis was performed using Microsoft Excel and IBM SPSS Statistics version 29.0 software (SPSS Inc., Chicago, IL, United States) for Windows. We used the Shapiro–Wilk test to determine whether the data were normally distributed. Continuous variables were expressed as mean ± standard deviation (SD) or median and interquartile range (IQR). Qualitative data were presented as counts and percentages (%). As mentioned previously, the whole cohort was subdivided into two groups according to the MIS-C severity class of GSATool: Group 1 (GSATool class I and class II) and Group 2 (GSATool class III and class IV). Both groups were compared by an independent samples *t*-test if the data were normally distributed and by the Wilcoxon signed rank test and Mann–Whitney *U* test for non-parametric data. The diagnostic accuracy of MIS-C biomarkers was compared using receiver operating characteristic (ROC) curves. Youden's index was used to determine the cutoff values. A *p*-value < 0.05 was considered significant.

### 2.5. Ethical consent

Permission to conduct the study was obtained from the Kaunas Regional Biomedical Research Ethics Committee (BE-2–27). This study was conducted in accordance with the Declaration of Helsinki and Good Clinical Practice Guidelines.

## 3. Results

### 3.1. General characteristics

In total, 43 children with a median age of 8 (5.2–10.9) years were included in the study. Only 13 pediatric patients matched GSATool classes III and IV and were assigned to Group 2. Older children had a more severe MIS-C course (*p* = 0.046). In total, 67.4% were boys, and a higher percentage of males was found in both severity groups. Half of the children were from the city (Table 1). More children with a lower MIS-C score were present in the Kaunas City area, but this was not significant (*p* = 0.587). Only seven children indicated a previous COVID-19 infection; however, data were lacking in the majority of the cases. Twenty children had previous contact with COVID-19-infected persons (relative, at school, etc.), and this did not differ significantly between the two groups. The median duration from contact to the first symptoms was 27.5 (25–30) days (Table 1), with no difference between cohorts (*p* = 0.253). Approximately half (48.8%) of the referred children were admitted to the PICU, with a median LOS of 2 (1–4) days in the PICU. More children were admitted in Group 2, but the difference was not significant (*p* = 0.078). Altogether, the median LOS was 10 (7–12) days. A significantly longer LOS was observed in more severe cases [9 (7.8–10.8) vs. 15 (10.9–19.6), respectively, *p* = 0.004] with no difference in the PICU LOS (*p* = 0.547) (Table 2).

**Table 1:** General characteristics of total MIS-C patients and according to the severity groups.

Number of patients included (*n*)			Group 1	Group 2	*p*-value
		Total (*n* = 43)	*n* = 30	*n* = 13	
**Median age (year) (IQR)**		**8** (**5.2–10.9)**	**7** (**6.9–8.8)**	**10** (**7.6–12.6)**	**0**.**046**
Age groups (year) (%)	<5	10 (23)	9 (30)	1 (7.7)	0.422
	5–10	18 (41.9)	12 (40)	6 (46.2)	
	10–15	13 (30.2)	8 (26.7)	5 (38.5)	
	>15	2 (4.7)	1 (3.3)	1 (7.7)	
Gender	Gender (male) (%)	29 (67.4)	21 (70)	8 (61.5)	0.587
Location	Region (%)	20 (46.5)	12 (40)	8 (61.5)	0.193
	City (%)	23 (53.5)	18 (60)	5 (38.5)	
SARS-CoV-2 infection	Positive PCR (%)	7 (16.3)	4 (13.3)	3 (23.1)	0.091
	Not detected (%)	5 (11.6)	5 (16.7)	0 (0)	
	No data (%)	31 (72)	21 (70)	10 (76.9)	
COVID contact	Positive (%)	20 (46.5)	16 (53.3)	4 (30.8)	0.203
	Negative (%)	21 (48.8)	13 (43.3)	8 (61.5)	
	No data (%)	2 (4.7)	1 (3.3)	1 (7.69)	
Median duration from contact to first MIS-C symptoms (day) (IQR)		27.5 (25–30)	5.63 (3.29–7.97)	4.46 (3.12–5.80)	0.253
Severity score (*n*, %)	0	15 (34.9)			
	I	6 (14.0)			
	II	9 (20.9)			
	III	10 (23.3)			
	IV	3 (7.0)			
Admitted to PICU (*n*) (%)		21 (48.8)	12 (40)	9 (69.2)	0.078
**Median LOS (day) (IQR)**	** **	**10** (**7–12)**	**9.30** (**7.81–10.79)**	**15.23** (**10.87–19.60)**	**0**.**004**
Median LOS in PICU (day) (IQR)		2 (1–4)	2.36 (1.27–3.46)	3.38 (1.65–5.10)	0.547

*n*, number; IQR, interquartile range; SD, standard deviation; PCR, polymerase chain reaction; SARS-CoV-2, severe acute respiratory syndrome coronavirus; COVID, coronavirus disease; MIS-C, multisystem inflammatory syndrome in children; PICU, pediatric intensive care unit; LOS, length of stay.

*p*-value < 0.05 was considered statistically significant. Statistically significant results are provided in bold.

**Table 2:** Symptoms and clinical features of total MIS-C patients according to the severity groups.

Symptoms and clinical features	Total, *n*	Severity group		*p*-value
	43	Group 1 (*n* = 30)	Group 2 (*n* = 13)	
Vomit, *n* (%)	21 (49)	14 (47)	7 (54)	0.665
Abdominal pain, *n* (%)	24 (56)	15 (50)	9 (70)	0.244
Diarrhea, *n* (%)	16 (37)	10 (33)	6 (46)	0.424
Obstipation, *n* (%)	2 (5)	2 (7)	0	0.342
Mesadenitis, *n* (%)	13 (30)	7 (23)	6 (46)	0.135
Lymphadenopathy, *n* (%)	7 (16)	5 (17)	2 (15)	0.917
Conjunctivitis, *n* (%)	25 (58)	17 (57)	8 (62)	0.766
Rash, *n* (%)	31 (72)	22 (73)	9 (69)	0.783
Sole desquamation, *n* (%)	4 (9)	3 (10)	1 (8)	0.811
Raspberry lips, tongue, *n* (%)	19 (44)	13 (43)	6 (46)	0.864
Pneumonia, *n* (%)	12 (28)	7 (23)	5 (38)	0.311
Pleuritis, *n* (%)	5 (12)	3 (10)	2 (15)	0.698
Bronchitis, *n* (%)	8 (19)	3 (10)	5 (38)	0.058
Coronary injury, *n* (%)	4 (9)	1 (3)	3 (23)	0.154
Other symptoms (neurological, nephrological, and articular), *n* (%)	9 (21)	6 (20)	3 (23)	0.312
**Shock, *n* (%)**	**4** (**9)**	**1** (**3)**	**3** (**23)**	**0**.**041**

*n*, number; IQR, interquartile range; MIS-C, multisystem inflammatory syndrome in children.

*p*-value < 0.05 was considered statistically significant. Statistically significant results are provided in bold.

### 3.2. Clinical features

On referral, most frequently, the children complained of gastrointestinal symptoms, rash, and conjunctivitis (Table 2). Twelve children had clinical features of pneumonia, and four presented with shock (Table 2). None of the symptoms or clinical features differed significantly between the severity groups, except for shock (*p* = 0.041). However, more severe MIS-C cases presented with abdominal pain and vomiting, and more cases had features of mesadenitis and bronchitis (Table 2).

### 3.3. Laboratory test results

Blood was drawn from all the patients for CBC, CRP, and other analyses of MIS-C biomarkers. Neu count was significantly higher in more severe MIS-C cases (Group 2) compared with that in Group 1 patients [10.4 (7.3–13.5) vs. 6.8 (5.4–8.2), respectively, *p* = 0.013] (Table 3). Other cell counts, Hgb, and PLT markers did not differ (Table 3). A significant increase in CRP levels was seen in more severe MIS-C cases (*p* = 0.027), along with other markers such as DD (*p* = 0.027), pro-BNP (*p* = 0.013), FR (*p* = 0.002), LDH (*p* = 0.032), and urea (*p* = 0.049) (Table 3).

**Table 3:** Laboratory test results of total MIS-C patients on admission and according to the severity groups.

Laboratory tests	Total	Severity group		*p*-value
CBC	(*n* = 43)	Group 1 (median, IQR)	Group 2 (median, IQR)	
WBC, 10^9^ cells/L	9.2 (6–12)	9.1 (7.5–10.8)	12.1 (8.6–15.5)	0.083
**Neu (×10**^9^ **cells/L)**	**7** (**4–11)**	**6.8** (**5.4–8.2)**	**10.4** (**7.3–13.5)**	**0**.**013**
Lymph (×10^9^ cells/L)	1.4 (0.5–1.6)	1.6 (1.1–2.1)	1 (0.6–1.4)	0.160
Hgb (g/L)	120 (111–126)	118 (113.8–122.4)	122 (113.7–130.8)	0.321
PLT (×10^9^ cells/L)	194 (102–303)	237 (174.3–299.2)	221 (129.2–313.2)	0.776
MPV (fl)	8.35 (7.2–9.4)	8.08 (7.61–8.56)	8.77 (8.25–9.28)	0.076
PCT (%)	0.17 (011–0.24)	0.18 (014–0.23)	0.21 (0.13–0.29)	0.538
PDW (%)	16.6 (16.3–17.0)	16.60 (16.42–16.77)	16.90 (16.57–17.23)	0.068
**CRP (*n* = 43) (mg/L)**	**145.8** (**100–222)**	**135.1** (**103.9–166.2)**	**199.9** (**145.7–254.1)**	**0**.**027**
ESR (*n* = 41) (mm/h)	27 (14–41)	28.66 (18.75–38.56)	34.31 (20.82–47.80)	0.498
PCT** (*n* = 38) (ng/ml)	1.58 (0–7.75)	4.81 (1.11–8.52)	12.41 (2.22–22.59)	0.071
PT (*n* = 42) (%)	28.4 (24–34)	29.13 (22.94–35.32)	35.92 (26.27–45.58)	0.216
APTT (*n* = 42) (s)	38 (34–43)	36.42 (31.96–40.89)	39.57 (35.28–43.86)	0.377
INR (*n* = 39)	1.2 (1.1–1.3)	1.16 (1.05–1.26)	1.25 (1.17–1.33)	0.247
**D-dimers (*n* = 41) (ng/ml)**	**3.1** (**2.0–4.5)**	**3.01** (**2.43–4.37)**	**4.78** (**3.42–6.14)**	**0**.**027**
Fibrinogen (*n* = 39) (g/L)	4.3 (2.6–5.4)	3.40 (2.43–4.37)	4.78 (3.42–6.14)	0.094
**Troponin I (*n* = 39) (ng/ml)**	**0.06** (**0.03–0.20)**	**0.13** (**0.01–0.24)**	**4.42** (**1.24–13.43)**	**0**.**002**
**Pro-BNP (*n* = 38) (**	**82** (**0–221)**	**85.84** (**44.03–127.65)**	**475.03** (**27.31–922.75)**	**0**.**013**
**Ferritin (*n* = 39) (mcg/L)**	**155** (**78–280)**	**150.56** (**101.39–199.72)**	**409.85** (**182.45–637.25)**	**0**.**002**
**LDH (*n* = 39) (U/L)**	**236** (**0–303)**	**174.73** (**123.91–225.55)**	**276.77** (**185.33–368.21)**	**0**.**032**
Albumin (*n* = 39) (g/L)	27 (0–31)	19.68 (12.96–26.40)	20.95 (12.05–29.84)	0.818
Creatine (*n* = 41) (µmol/L)	39 (31–47)	39.66 (32.43–46.98)	47.00 (33.49–60.51)	0.283
**Urea (*n* = 40) (U/L)**	**4.0** (**2.8–4.8)**	**3.70** (**3.21–4.20)**	**4.87** (**3.38–6.36)**	**0**.**049**
ALT (*n* = 40) (IU/L)	26 (18–43)	35.18 (24.46–45.90)	32.25 (23.64–40.86)	0.730
AST (*n* = 40) (U/L)	36 (28–49)	40.68 (31.44–49.92)	41.83 (34.20–49.47)	0.875
GGT (*n* = 38) (IU/L)	18.5 (12.5–39)	31.65 (15.26–48.05)	39.58 (18.70–40.46)	0.867

CBC, complete blood count; WBC, white blood cell; Lymph, lymphocytes; Mono, monocytes; Hgb, hemoglobin; PLT, thrombocytes; MPV, mean platelet volume; PCT, plateletcrit; PDW, platelet distribution width; CRP, C-reactive protein; ESR, erythrocyte sedimentation rate; PCT**, procalcitonin; PT, prothrombin time; APTT, activated partial thromboplastin time; INR, international normalized ratio; pro-BNP, NT-proB-type natriuretic peptide; LDH, lactate dehydrogenase; ALT, alanine transaminase; AST, aspartate aminotransferase; GGT, gamma-glutamyl transferase; MIS-C, multisystem inflammatory syndrome in children.

*p*-value < 0.05 was considered statistically significant. Statistically significant results are provided in bold.

### 3.4. Prediction of MIS-C severity

We analyzed different marker combinations for predicting MIS-C severity. First, routine blood biomarkers were investigated following the MIS-C guidelines. All combinations were significant in predicting a severe course of MIS-C (Table 4). We observed that the combination of CRP with Neu with a cutoff value of 0.834 and area under the ROC curve (AUC) of 0.774 had a specificity of 92.3%; however, the sensitivity was 60% (Table 4). When biomarkers were added stepwise, sensitivity and specificity increased, with the best results obtained when all the biomarkers according to the MIS-C diagnostic guidelines were included (Table 4).

**Table 4:** Analysis of different laboratory parameters in MIS-C severity prediction.

Laboratory parameters	Cutoff value	Youden's index	AUC (95% CI)	Sensitivity %	Specificity %	*p*-value
CRP + Neu	0.834	0.523	0.774 (0.636–0.912)	60	92.3	<0.001
CRP + Neu + PCT**	0.768	0.494	0.728 (0.566–0.890)	57.7	91.7	0.006
CRP + Neu + PCT** + DD	0.623	0.47	0.800 (0.647–0.953)	80	67	<0.001
CRP + Neu + PCT** + DD + FB	0.552	0.614	0.860 (0.736–0.984)	86.4	75	<0.001
CRP + Neu + PCT** + DD + FB + pro-BNP	0.774	0.69	0.902 (0.801–1.002)	77.3	91.7	<0.001
CRP + Neu + PCT** + DD + FB + pro-BNP + FR	0.807	0.773	0.917 (0.823–1.012)	77.3	100	<0.001
CRP + Neu + PCT** + DD + FB + pro-BNP + FR + LDH	0.613	0.861	0.965 (0.909–1.022)	95.2	90.9	<0.001
CRP + Neu + PCT** + DD + FB + pro-BNP + FR + LDH + U	0.500	1	1.000 (1.000–1.000)	100	100	<0.001
CRP + Neu + Lymph + FR + pro-BNP + PCT**	0.836	0.75	0.924 (0.840–1.009)	75.0	100	<0.001

CRP, C-reactive protein; Neu, neutrophils; PCT**, procalcitonin; DD, D-dimer; FB, fibrinogen; pro-BNP, NT-proB-type natriuretic peptide; FR, ferritin; LDH, lactate dehydrogenase; U, urea; Lymph, lymphocyte; AUC, area under the ROC curve; CI, confidence interval.

*p*-value < 0.05 was considered statistically significant.

### 3.5. PLTs and PLT parameters

We analyzed the potential of PLT, MPV, PCT, and PDW in predicting MIS-C severity. With a cutoff value of 0.836 for the combination of these markers (without any additional CBC, CRP, and other biomarkers), the sensitivity and specificity for predicting MIS-C were 80.8% and 91.7%, respectively (AUC of 0.924; 95% CI of 0.940–1.009; *p* < 0.001) (Table 5). The inclusion of those markers along with the routine blood biomarkers enhanced their ability to predict MIS-C severity, increasing both sensitivity and specificity (Table 5).

**Table 5:** Analysis of platelet marker combinations with different laboratory parameters in MIS-C severity prediction.

Laboratory parameters	Cutoff value	Youden's index	AUC (95% CI)	Sensitivity %	Specificity %	*p*-value
PLT + MPV + PCT + PDW	0.716	0.725	0.901 (0.803–0.998)	80.8	91.7	<0.001
PLT + MPV + PCT + CRP	0.810	0.615	0.827 (0.760–0.984)	61.5	100	<0.001
PLT + MPV + PCT + PCT**	0.687	0.773	0.917 (0.821–1.014)	77.3	100	<0.001
PLT + MPV + PCT + PDW + pro-BNP	0.667	0.71	0.929 (0.845–1.012)	85.7	85.3	<0.001
PLT + MPV + PCT + PDW + FR	0.759	0.87	0.949 (0.877–1.020)	87.0	100	<0.001
PLT + MPV + PCT + PDW + LDH	0.642	0.83	0.906 (0.794–1.018)	91.3	91.7	<0.001
PLT + MPV + PCT + PDW + U	0.788	0.75	0.886 (0.779–0.994)	75.0	100	<0.001
PLT + MPV + PCT + PDW + FB	0.616	0.697	0.917 (0.826–1.007_	86.4	83.3	<0.001
PLT + MPV + PCT + PDW + LDH + FR	0.567	0.814	0.944 (0.855–1.032)	90.5	90.9	<0.001
PLT + MPV + PCT + PDW + LDH + pro-BNP	0.500	1	1.000 (1.000–1.000)	100	100	<0.001

PLT, platelet; MPV, mean platelet volume; PCT, plateletcrit; PDW, platelet distribution width; CRP, C-reactive protein; PCT**, procalcitonin; pro-BNP, NT-proB-type natriuretic peptide; FR, ferritin; LDH, lactate dehydrogenase; U, urea; FB, fibrinogen; MIS-C, multisystem inflammatory syndrome in children; AUC, area under the ROC curve; CI, confidence interval.

*p*-value < 0.05 was considered statistically significant.

## 4. Discussion

MIS-C is a cytokine storm–caused condition and requires immediate and aggressive treatment in severe cases. We evaluated a total of 43 MIS-C cases admitted and treated in our university hospital. All of the patients were previously healthy with no comorbidities. Children presented with a variety of symptoms, and half of them required treatment in PICU. Although a single PLT biomarker did not differ between the severity groups, we found that the combination of PLT count and PLT indices had the potential to predict MIS-C severity.

The risk of MIS-C has been influenced by the spread of different variants of SARS-CoV-2 virus. Various studies have identified that the incidence of MIS-C was markedly lower during the Omicron wave, compared with earlier variants of the virus (31–33). According to our study, nearly half (48%) of the MIS-C cases emerged following the initial wave of COVID-19 in Lithuania. Unfortunately, most of our patients did not undergo polymerase chain reaction (PCR) for SARS-CoV-2 before developing MIS-C. Thus, we relied on epidemiological anamnesis of confirmed COVID-19 illness in the close contacts. The median time to the MIS-C onset was around 4 weeks (27.5 days), which corresponds to the MIS-C outbreaks observed in the majority of previous studies (8, 10, 11, 26, 29, 34–36).

MIS-C is characterized by fever, overwhelming systemic inflammation, hypotension, and cardiac dysfunction. Recent studies show that the severity of this condition can range from mild cases with lower inflammation markers to the most severe cases presenting with shock and MAS-like features (6, 37, 38). Our analyzed cohort of children also covered this wide spectrum of presentations and severities of MIS-C. The mean age of 8 years and male gender predominance (67.4%) found in our study were in line with the data from most MIS-C studies (6, 10, 11, 13, 26, 29, 34–36, 39). Moreover, the leading clinical manifestations of MIS-C were rash, gastrointestinal symptoms, and conjunctivitis (Table 2). In general, all clinical signs were consistent with previously published MIS-C studies and case reports of predominant abdominal symptoms, rash, and cardiovascular involvement (6, 39–41). Various data show that approximately 30%–60% of patients present with symptoms of shock and the need for treatment in the PICU (10, 26, 29, 34). In our case, only four children had symptoms of shock. In general, by analyzing our data, we retrospectively used a specific scoring method to identify the most severe patients and investigate the possible prognostic factors that could be used in early risk evaluation. We used the composite severity score (further GSATool) proposed by Brisca et al. (30). The researchers demonstrated that by using this multistep early risk evaluation (GSATool) and aggressive therapeutic approach, all 23 patients included in their study avoided admission to the PICU, invasive mechanical ventilation, extracorporeal circulatory and respiratory support, or administration of inotropic drugs. Most of our analyzed MIS-C patients fell into the less severe group (classes I and II according to the GSATool) (*n* = 30), and the majority of the cases did not differ in clinical symptoms or features at presentation, except for signs of shock (*p* = 0.041).

Admission to the PICU is one of the most evaluated outcomes for MIS-C severity. Systematic reviews conducted by Hoste et al. and Radia et al. have reported that approximately 68%–74% of MIS-C patients need treatment in the intensive care unit, and more than half of them (56.3%–77%) present with shock (13, 38). Another group of patients deteriorated during the disease course and were transferred to the PICU mainly because of the need for inotropic treatment (26, 38). In our study, nearly half of the children (48.8%) were admitted to the PICU. We observed that children were more frequently treated in the PICU if they scored higher according to the GSATool, although this was not statistically significant. Interestingly, 40% of Group 1 patients were admitted to the PICU despite the absence of the need for respiratory or cardiovascular support, which suggests that referral to the PICU may be influenced by the subjective decision of different physicians and the lack of specific severity (and/or outcome) prognostic factors in the early stage of the disease. Similar data were reported by Kaidar et al. in a retrospective multicenter study where one-third of the cases were treated in the PICU without the need for inotropic medications or vasopressors (26). The researchers hypothesized that admission to the PICU may not be the best outcome measure for MIS-C severity.

Nevertheless, the recovery from the severe condition is quite fast as the median duration in hospital in most of the studies does not exceed 10 days (11, 13, 27, 36, 42). We found similar results, with a short LOS in the PICU with a median of 2 days and an overall LOS in the hospital of a median of 10 days. The total LOS was mostly influenced by the severity group and admission to the PICU.

Regarding the variety of presentation signs of MIS-C, all patients underwent a screening panel of biomarkers according to the existing guidelines. Our findings of high Neu count and high values of CRP, DD, FR, and pro-BNP as risk factors for severe MIS-C (Table 3) are comparable to those of several previous studies (10, 26, 34, 36, 38, 43, 44). In one of the largest cohorts of 1,080 MIS-C patients, Abrams et al. determined that the odds of severe MIS-C increased significantly by two times with increased CRP, FR, and DD levels, and MIS-C was five times more likely if the level of pro-BNP was above 2,000 pg/ml (34). A similar importance of pro-BNP was reported in the study performed by Kaidar et al., where an increasing amount of pro-BNP could increase the risk of severe MIS-C by 8.4 times upon reaching levels above 8,000 ng/L (26). The significance of cardiac biomarkers in the early prediction of MIS-C in pediatric patients with COVID-19 has been determined in several studies (44, 45). Gullu et al. identified that a pro-BNP value of 282 ng/L or more alone had a striking sensitivity of 100% and a specificity of 93% with an AUC of 0.985, while an increase in troponin I was less sensitive (60%) but more specific (99.2%, AUC of 0.794) (44). Furthermore, a study from Israel has shown the associations of hemoglobin level ≤95 g/L [odds ratio (OR) 3.356 (1.06–10.61)], PLT count <150 K/ml [OR 4.26 (1.40–12.96)], and CRP value ≥200 mg/L [OR 4.44 (1.45–13.58)] with more severe MIS-C courses (26). We did not find any association of hemoglobin, lymphocyte, and PLT counts with the different severity groups, which was reported earlier (6, 11, 26, 27, 34). Certainly, our cohort was small, and some other studies have addressed higher number of patients. Moreover, we did not include patients older than 18 years of age in our analysis. This population is shown to be associated with more severe MIS-C (46). Nevertheless, in our study, we observed that older patients presented with more severe MIS-C features compared with younger ones [7 (6.9–8.8) vs. 10 (7.6–12.6), respectively, *p* = 0.046]. As expected, all the routine inflammatory markers used for the diagnosis of MIS-C could predict the severity of the condition in our cohort. The combination of CRP and Neu alone was highly specific (92.6%) but was not sensitive enough (60%) to predict the severity group. In addition, the combination of all biomarkers in the MIS-C diagnostic panel showed the highest sensitivity and specificity (Table 4). However, not all MIS-C routine biomarkers are freely available in primary care or small regional hospitals (43). Thus, a simpler and more easily obtainable prediction model of disease severity would enable faster referral of suspected severe cases to specialized centers or prompt prescription of specific treatment.

The need for new biomarkers for the evaluation of severe conditions has raised interest in the involvement of PLTs in immunity and their activation signs. Recently, a clinically well-known prognostic factor of thrombocytopenia in severe bacterial infections was supplemented by changes in the MPV and PDW as evidence of innate and adaptive immune response activation (18). Moreover, Barrett et al. found that reticulated PLT count, larger size, and immaturity in SARS-CoV-2 infection were associated with more severe disease and all-cause mortality (25). Furthermore, in severe cases of MIS-C that needed inotropic support or a longer stay in the PICU, the PLT count was lower and DD and FB levels were higher, indicating upregulated coagulation (6, 26–29, 34). PVT volume index (PVI) values as prognostic indices have been demonstrated in several studies of COVID-19 patients (47); however, data on MIS-C are limited. In a retrospective single-center study of 64 MIS-C patients, Alkan et al. showed that MPV was significantly higher in the most severe patient group (39). However, other parameters (PDW and PCT) could not differentiate between severity groups. In our patient cohort, none of the PLT indices alone differed between the severity groups. MPV and PDW tended to be higher in patients with more severe disease, but the difference was not significant (*p* = 0.076 and *p* = 0.068, respectively). Interestingly, we found that a combination of these biomarkers (PLT, MPV, PCT, and PDW) with a cutoff value of 0.716 could predict MIS-C severity with a sensitivity of 80.8% and specificity of 91.7% (AUC of 0.901; 95% CI of 0.803–0.998; *p* < 0.001), demonstrating the potential of a fast and reliable composite biomarker in clinical practice. A routine clinical blood test using automated hematology analyzers can easily evaluate PVI and count the PCT—the volume occupied by PLTs in the blood as a percentage; thus, it could be easily applied in settings without the possibility of testing biomarkers, such as pro-BNP. PLTs have been shown to have the potential to differentiate between bacterial and viral diseases (48), which could help clinicians to differentiate between sepsis and viral-induced sequelae if MIS-C is suspected. Definitely, further studies are needed. Moreover, more research including healthy children, children with other viral diseases, and children with real KD should be included in a broader analysis to justify the use of PLT markers in clinical practice.

In conclusion, our study is the first in Lithuania to describe clinical and laboratory parameters associated with MIS-C severity, highlighting the significant role of PLTs in MIS-C pathogenesis and severity. PLTs and PLT index measurements in routine laboratory analyses may be helpful in predicting MIS-C severity. However, further prospective and follow-up studies are warranted.

## Data Availability

The raw data supporting the conclusions of this article will be made available by the authors, without undue reservation.
